# Obesity as a Possible Risk Factor for Progression from Monoclonal Gammopathy of Undetermined Significance Progression into Multiple Myeloma: Could Myeloma Be Prevented with Metformin Treatment?

**DOI:** 10.1155/2021/6615684

**Published:** 2021-01-18

**Authors:** Ademar Dantas da Cunha Júnior, Dalila Luciola Zanette, Fernando Vieira Pericole, Sara Teresinha Olalla Saad, José Barreto Campello Carvalheira

**Affiliations:** ^1^Division of Oncology, Department of Internal Medicine, Faculty of Medical Sciences, State University of Campinas (UNICAMP), Campinas, SP, Brazil; ^2^Hematology and Oncology Clinics, Cancer Hospital of Cascavel, União Oeste de Estudos e Combate ao Câncer (UOPECCAN), Cascavel, PR, Brazil; ^3^Department of Internal Medicine, State University of Western Paraná (UNIOESTE), Cascavel, PR, Brazil; ^4^Laboratory for Applied Science and Technology in Health, Carlos Chagas Institute (ICC), Oswaldo Cruz Foundation (Fiocruz), Rio de Janeiro, Brazil; ^5^Hematology and Blood Transfusion Center, University of Campinas (UNICAMP), Campinas, SP, Brazil

## Abstract

Obesity is increasingly associated with the transformation of monoclonal gammopathy of undetermined significance (MGUS) into multiple myeloma **(**MM). Obesity, MGUS, and MM share common etiopathogenesis mechanisms including altered insulin axis and the action of inflammatory cytokines. Consistent with this interconnection, metformin could predominantly exert inhibition of these pathophysiological factors and thus be an attractive therapeutic option for MGUS. Despite the possible clinical significance, only a limited number of epidemiological studies have focused on obesity as a risk factor for MGUS and MM. This review describes multiple biological pathways modulated by metformin at the cellular level and their possible impacts on the biology of MGUS and its progression into MM.

## 1. Introduction

Brazilian studies have revealed a remarkable prevalence of diabetes [1], obesity [2], and cancer [3] in adults over time and with a progressive expansion in the last years. Diabetes mellitus type 2 (DM2) and cancer share many risk factors, especially obesity and metabolic syndrome, with potential biological connections between the two diseases, as meta-analysis data from prospective cohort studies suggest a modest but consistent direct effect of body mass index (BMI) on the incidence of lymphoma, multiple myeloma, increased risk of leukemia in adults [4–13], increased risk of monoclonal gammopathy of undetermined significance (MGUS) transformation into MM [5, 12, 14], and increased risk of death in MM patients [15]. Data linking obesity to myeloma are the most convincing among hematologic malignancies, based on their replication in most studies [16–18]. Although metformin has been shown to modulate multiple biological pathways at the cellular level [19], few studies have focused on the effects of metformin on the biology of MGUS and the protection from MM transformation. Although MM therapeutic approaches conferred remarkable progress in the last decade, the mortality of this disease is still considerably high. Moreover, the therapeutic approaches available following relapse are not feasible in low-income countries and are financially costly for the public health system [20, 21]. Therefore, low-cost drugs that have a positive impact on prevention should be carefully studied as an attempt to avoid MGUS into MM progression.

## 2. Discussion

### 2.1. Obesity in MM

Three percent of the population above 50 and 5% above 70 years of age has MGUS [22]. The incidence of progression into myeloma, lymphoma, or amyloidosis among patients with MGUS is about 1% per year [22]. MGUS and MM share common risk factors that include occupational exposure to benzene and pesticides [23, 24], advanced age [25], African ancestry [26, 27], and male gender [25]. Recent evidence suggests common genetic susceptibility to MM and MGUS [28–30] but still require further studies. It should be noted that, in recent years, there is growing evidence that obesity acts as a risk factor for the occurrence of MM [4, 16–18, 31, 32]. In addition, obesity affects the transformation of MGUS into MM [12, 33, 34]. From the epidemiological point of view, there are prospective as well as meta-analyses data that support the association between increased BMI and increased risk of developing hematological malignancies [4, 9, 15, 35–37]. However, the role of confounder factors for this association such as dietary habits, physical activity, and/or antidiabetic therapy is largely unknown [38].

The modified insulin signaling axis [32–35], the release of adipokines [36–41], and lastly, low-grade chronic inflammation [34, 42] are plausible molecular mechanisms for the association between obesity and hematological neoplasms. Leptin in combination with bone marrow microenvironment cytokines, for example, interleukine-6 (IL-6), insulin-like growth factor-1 (IGF-1), vascular endothelial growth factor (VEGF), and TNF-alpha lead to adipocyte differentiation of bone marrow mesenchymal stromal cells (BM-MSCs), thus contributing to an increase in the adipose marrow tissue [39, 40]. Therefore, all these mediators negatively affect hematopoiesis and subsequently lead to the remodeling of the bone marrow microenvironment that is observed in obesity which favors the growth of cells neoplasms in bone marrow (BM) [37–39]. Likewise, IGF-1 and IL-6 receptors show JAK-independent synergistic effects on the induction of MM cell growth [41]. IGF-1 is an endocrine factor produced and secreted by bone marrow stromal cells (BMSC), bone endothelial cells, and osteoblasts that promotes the homing, growth, and survival of myeloma cells in the bone marrow environment, both dependent and independent of IL-6 [42]. The binding of the ligand to the IGF-1 receptor (IGF-1R) via the tyrosine kinase (TK) domain activates the phosphoinositide 3-kinases/protein kinase B (PI3K/Akt) [43, 44] and mitogen-activated protein/extracellular signal-regulated kinases (MAPK/MEK/ERK) signaling pathways [43, 44]. Indeed, IGF-1 mediates multiple effects on MM cells through different signal transduction pathways. IGF-1 binding to IGF-1R triggers tyrosine kinase, resulting in activation of PI3K/Akt and RAS/RAF/MEK-ERK. PI3K/Akt is involved mainly in the antiapoptotic effects, whereas the MEK-ERK regulates cell cycle and proliferation. In addition to these main pathways, IGF-IR is able to activate the Janus kinases/signal transducer and activator of transcription proteins (JAK/STAT) pathway and Wnt and nuclear factor kappa-light-chain-enhancer of activated B cells (NF-*κ*B) signaling [45] IGF-1 induces VEGF production in MM cells through the MEK/ERK pathway, which increases angiogenesis in the BM [46, 47]. IL-6 is a cytokine with pleiotropic effects in hematopoietic and nonhematopoietic cells [39] with prognostic value in MM [48, 49]. IL-6 is mostly secreted by the BMSC, and its production and secretion are enhanced by the adhesion between MM cells and BMSC, via NFkB [39]. VEGF and IL-6 show mutual stimulation in MM, in which VEGF acts as a paracrine mediator that supports MM cell growth through the increase of IL-6 secretion by BMSCs and microvascular endothelial cells. IL-6, in turn, may stimulate VEGF secretion in a subset of myeloma cells, indirectly promoting angiogenesis [50, 51]. Following binding to its receptor, IL-6 is able to trigger the activation of the MEK/MAPK [52], JAK/STAT [53], and PI3K/AKT signaling pathways [54].

Another essential signaling pathway that correlates with the development of myeloma bone disease (MBD) is the receptor activator of nuclear factor *κ*B (RANK)/ligand of RANK (RANKL) pathway, which stimulates the signals via NF‐*κ*B and MAPK pathway providing the development, maturation, and differentiation of osteoclast precursors [55]. These factors stimulate cell surface receptors and signaling through JAK/STATs, MAPK, and PI3K molecules frequently dysregulated in neoplastic cells [56].

Obesity and myeloma share signaling pathways that upregulate insulin, IGF-1, leptin, and inflammatory cytokines, raising the risk of malignant transformation [57, 58]. Consonant with this, a drug that potentially inhibits these pathways could be interesting as a therapeutic alternative in MGUS consequently being associated with a lower risk to develop MM [34]. The malignant transformation of a post-GC B cell or plasma cell into MGUS and consequently into MM requires both an initiating genetic event and multiple secondary genetic events [59, 60]. The malignant evolution of MGUS is mediated by structural and functional alterations of the tumor-associated stromal cells, producing in BM microenvironment that acts as an essential partner in carcinogenesis and hence can be a new target for therapy in early disease stages [59].

### 2.2. Metformin as a Preventive Drug in MM

The Diabetes Prevention Program (DPP) study showed that metformin constantly reduced body weight over time, which could explain the diabetes prevention effects of the drug. Metformin also significantly improved fasting insulin and proinsulin and other adiposity parameters such as BMI, waist circumference, and waist-hip ratio [61].

**Figure 1:**
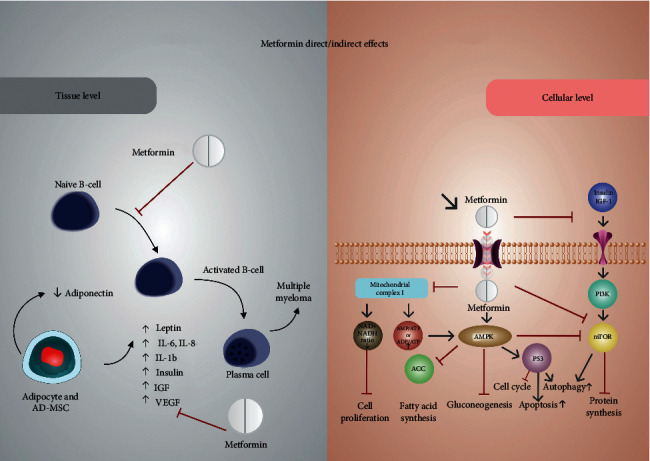
Figure modified from [32]. Overview of cellular and tissue mechanisms of metformin on inhibiting plasm cell growth. Metformin inhibits at tissue level: the inflammation caused by increases in circulating levels of leptin, insulin, IGF, IL-6, IL-1, and VGFR, driving by adipocyte differentiation of bone marrow mesenchymal stromal cells (AD-MSCs); and a cellular level inhibits mitochondria complex I, stimulates the adenosine monophosphate-activated protein kinase (AMPK) signaling pathway, and/or inhibits the insulin signaling pathway. ACC, acetyl-CoA carboxylase; EMT, epithelial-mesenchymal transition; IGF, insulin growth factor; IGF-1, insulin-like growth factor-1; IGF-1R, insulin-like growth factor-1 receptor; IR, insulin receptor; IL-1, interleukin 1; IL-6, interleukin-6, OCT1, organic cation transporter 1; VGFR, endothelial growth factor.

Epidemiological and preclinical research studies indicate that metformin is a potential therapeutic target in patients with leukemia [62, 63], lymphomas [63–65], and multiple myeloma [33, 34, 66–68]. It is expected that the diverse pleiotropic effects of the drug act on multiple targets, specifically in myeloma, in which there is a strong interaction between the clonal plasma cells and the BM microenvironment [69] (Figure 1).

Metformin might conduct to systemically reduce the levels of proinflammatory soluble mediators (e.g., IL-6 and IGF-1) [70]. A recent study showed metformin specifically decreased IL-6R expression, which is mediated via AMPK, mTOR, as well as displaying an isolated and synergistic effect with common antimyeloma drugs. Intriguingly, IL-6R can serve as a biomarker for metformin action in multiple myeloma [70]. Besides, it promotes appropriate direction and intensity, antitumor immunity-related metabolic checkpoints not solely in T cells, cancer cells, and associated immune suppressor cells of the microenvironment; furthermore, it might interfere with the gut microbiota and its systemic impacts on body metabolism [71].

Finally, metformin influences bone turnover because it activates AMP-activated protein kinase (AMPK), which, in turn, acts as a negative regulator of RANKL in the differentiation of osteoclasts. Furthermore, metformin might also significantly suppress bone resorption [72, 73].

New epidemiological and preclinical research points to metformin as a potential therapeutic target in patients with multiple myeloma [5, 12, 33, 34, 68]. Although epidemiological studies have reasonably and consistently reported reduced MM incidence and/or mortality in diabetic patients who receive metformin [74–78], many sampled their case study retrospectively from the hospital or clinical registries rather than population-based registries, restricting external validity and inserting potential selection biases. Some studies did not exclude individuals with a prior cancer diagnosis, thus presenting a possible reverse causation bias. Many studies included patients exposed to various treatments for diabetes, complicating the analysis of metformin associations. Self-reporting of crucial data such as concomitant medication use and cancer risk factors such as obesity, tobacco use, and family history may have provided exposure biases. Moreover, research of metformin benefit markers will demand to include essential host factors such as circulating insulin and glucose levels, obesity, and expression of OCT1 receptors in the liver and the tumor [79]. Additionally, tumor cells' characteristics such as receptors (insulin/IGF1) [57, 80] and pathway proteins (PI3K/mTOR, LKB1, and TSC2) [81, 82] expression might potentially mediate these indirect, host-mediated effects and any direct effects that are extremely important.

Currently, there are a reduced number of epidemiological studies focused on obesity as a risk factor for MGUS and MM, notwithstanding the potential clinical importance of obesity in myeloma [83]. Obesity and DM2 are risk factors for myeloma that make metformin a potential protective therapy in the evolution of the natural history of the disease [12, 34].

## 3. Conclusion

Assuming that 3% of adults over the age of 50 have MGUS and the increasing numbers of diabetes and obesity in the world population, less toxic approaches are needed to minimize the chances of MGUS to MM progression. Antidiabetic drugs such as metformin are low-cost and safe, and studies have demonstrated their potentially protective roles in cancer, MM, and MGUS to MM progression. Interventions with minor results may have a meaningful influence on the cumulative disease load. Considering the pleiotropic effects (direct and indirect) of metformin on the bone marrow milieu, it is essential to investigate the mechanisms involved in the preventive effects of the drug in the progression of multiple myeloma, in order to indicate its proper use in this context.

## Data Availability

The data used to support the findings of this study are available from the corresponding author upon request.
